# Supplementary material for: In vivo mitochondrial base editing via adeno-associated viral delivery to mouse post-mitotic tissue
Source: Nat Commun. 2022 Feb 8;13:750. doi: 10.1038/s41467-022-28358-w (PMC8825850; doi:10.1038/s41467-022-28358-w)
Supplement: Supplementary file 1 — Supplementary Information [file 41467_2022_28358_MOESM1_ESM.pdf]

Supplementary Information

In vivo mitochondrial base editing via adeno-associated viral delivery to mouse post-mitotic tissue

Pedro Silva-Pinheiro[1], **Pavel A. Nash**[1], **Lindsey Van Haute**[1], **Christian D. Mutti**[1], **Keira Turner**[1] and **Michal Minczuk**[1*]

[1] MRC Mitochondrial Biology Unit, University of Cambridge, Cambridge, UK

*Corresponding author:

Tel: +44 1223 252750; E-mail: michal.minczuk@mrc-mbu.cam.ac.uk

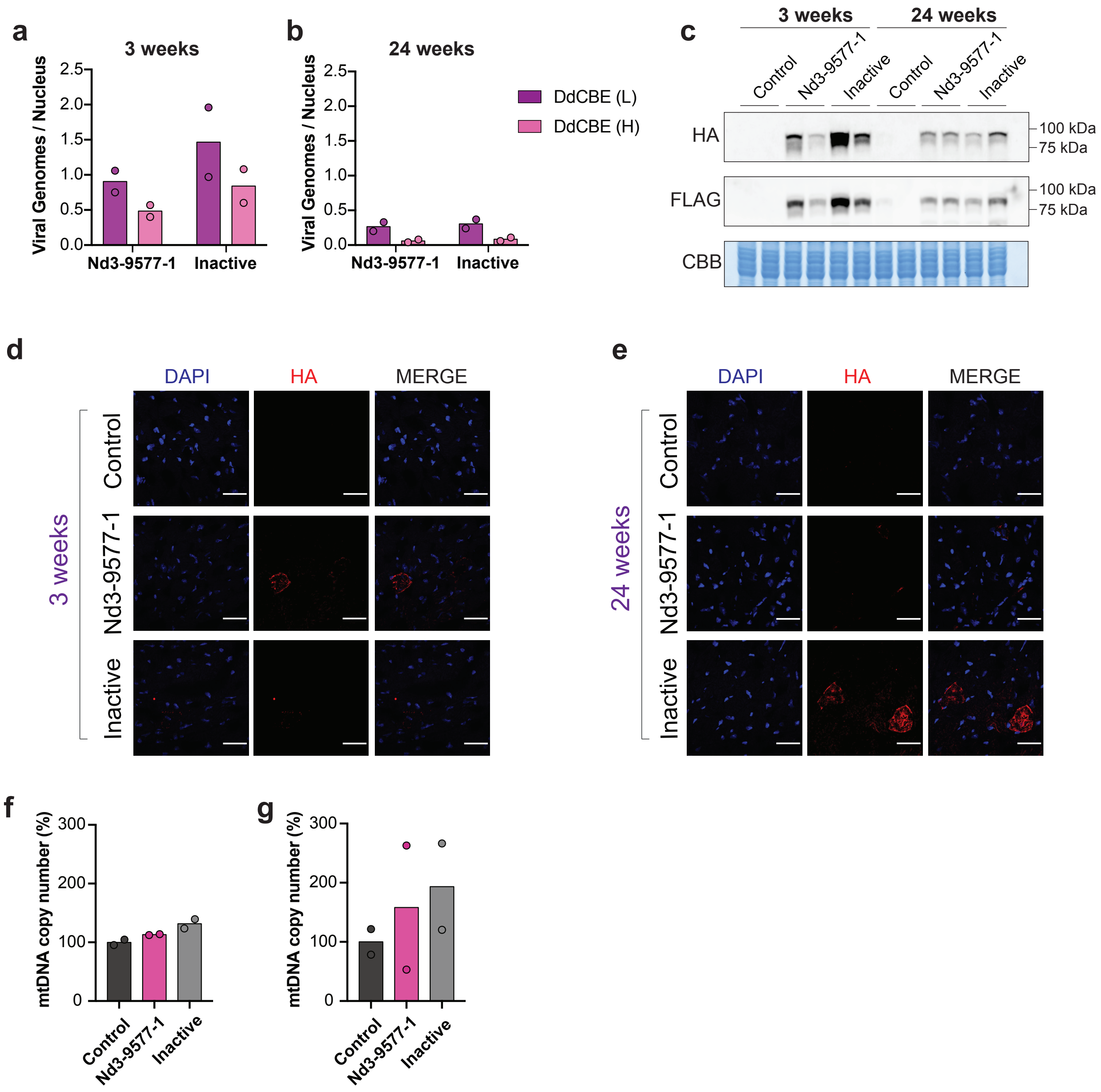

Supplementary Fig. 1 | Characterization of mouse hearts upon adult-delivery of AAV-DdCBEs. **a,b.** Real-Time qPCR quantification of AAV-DdCBE (L) and AAV-DdCBE (H) viral genomes per nucleus in hearts of adult-injected mice at 3 weeks (a) or 24 weeks (b) post-injection of either the catalytically active or inactive DdCBE-Nd3-9577-1 AAV pair. Bars represent the mean (n=2). Source data are provided as a Source Data file. **c.** Western blot analysis of the levels of HA-tagged DdCBE (L) and Flag-tagged DdCBE (H) in hearts of adult-injected mice at 3 weeks or 24 weeks post-injection of either the catalytically active or inactive DdCBE-Nd3-9577-1 AAV pair and vehicle injected mice (control). Coomassie Brilliant Blue (CBB) was used as loading control. Images representative of n=2 independent biological replicates. Each lane represents a sample from an individual mouse. **d,e.** Immunohistochemistry analysis of the HA-tagged DdCBE (L) in hearts of adult-injected mice at 3 weeks (d) or 24 weeks (e) post-injection of either the catalytically active or inactive DdCBE-Nd3-9577-1 AAV pair and vehicle injected mice (control). Scale bars: 30 µm. **f,g.** Real-Time qPCR relative quantification of mtDNA copy number in hearts of adult-injected mice at 3 weeks (f) or 24 weeks (g) post-injection of either the catalytically active or inactive DdCBE-Nd3-9577-1 AAV pair. Bars represent the mean (n=2). Source data are provided as a Source Data file.

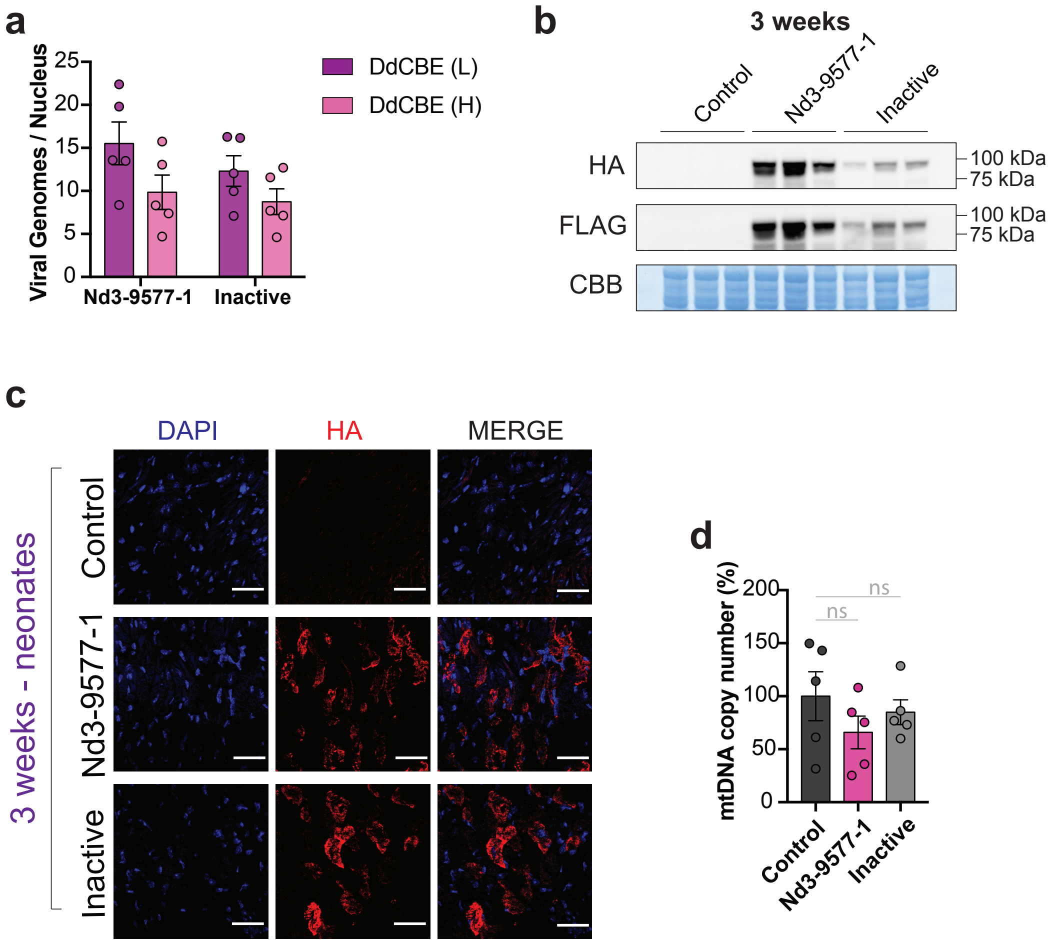

Supplementary Fig. 2 | Characterization of mouse hearts upon neonatal-delivery of AAV-DdCBEs.
a. Real-Time qPCR quantification of AAV-DdCBE (L) and AAV-DdCBE (H) viral genomes per nucleus in hearts of neonate-injected mice at 3 weeks post-injection of either the catalytically active or inactive DdCBE-Nd3-9577-1 AAV pair. Bars represent the mean and error bars represent ± SEM (n = 5). Source data are provided as a Source Data file. **b.** Western blot analysis of the levels of HA-tagged DdCBE (L) and Flag-tagged DdCBE (H) in hearts of neonate-injected mice at 3 weeks post-injection of either the catalytically active or inactive DdCBE-Nd3-9577-1 AAV pair and vehicle injected mice (control). Coomassie Brilliant Blue (CBB) was used as loading control. Images representative of n=3 independent biological replicates. **c.** Immunohistochemistry analysis of the HA-tagged DdCBE (L) in hearts neonate-injected mice at 3 weeks post-injection of either the catalytically active or inactive DdCBE-Nd3-9577-1 AAV pair and vehicle injected mice (control). Scale bars: 30 µm. **d.** Real-Time qPCR relative quantification of mtDNA copy number in hearts of neonate-injected mice at 3 weeks post-injection of either the catalytically active or inactive DdCBE-Nd3-9577-1 AAV pair. Bars represent the mean and error bars represent ± SEM (n = 5). Ordinary one-way ANOVA with Dunnett´s test: not significant - ns (Control vs Nd3-9577, P-value = 0.3142; Control vs Inactive, P-value = 0.7703). Source data are provided as a Source Data file.

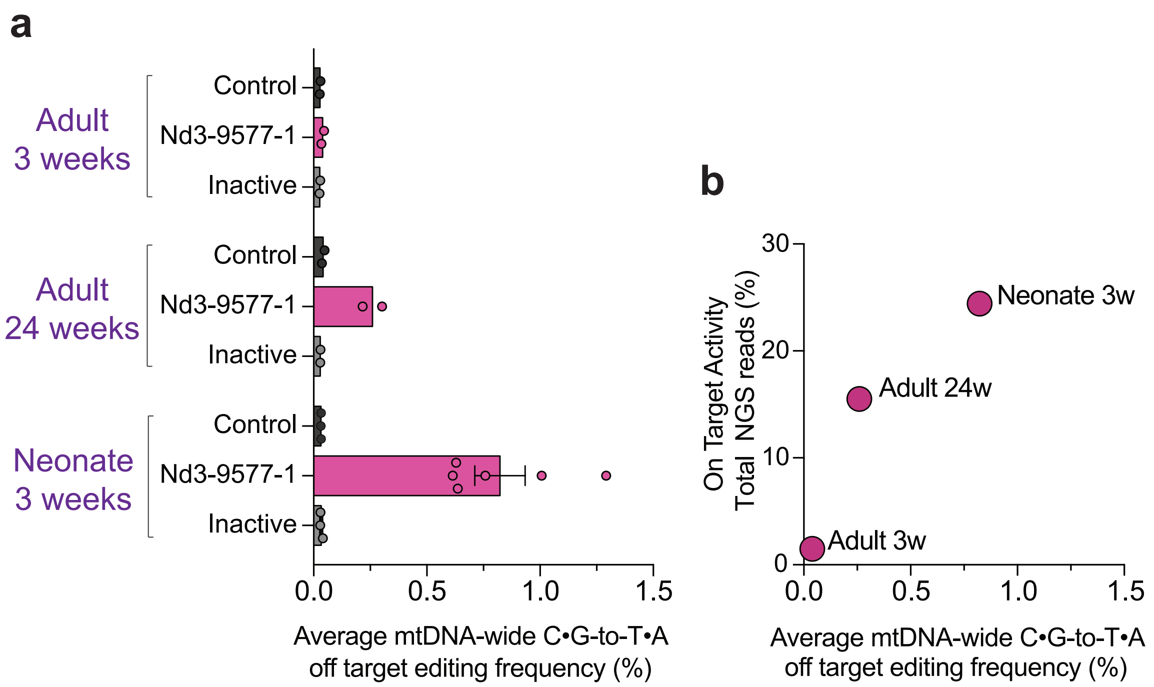

Supplementary Fig. 3 | Assessment of mtDNA-wide off-target activity *in vivo* upon delivery of AAV-DdCBEs.
a. NGS analysis of the average mtDNA-wide C·G to T·A off-target editing frequency by AAV-DdCBEs in adult mouse heart at 3 weeks or 24 weeks post-injection and in neonatal mouse hearts at 3 weeks post-injection. Each mouse was injected with either the catalytically active or inactive DdCBE-Nd3-9577-1 AAV pair or vehicle (control). Bars represent the mean and error bars represent ± SEM (n=2 for all experiments in adults; n=3 for vehicle and inactive base editor controls in neonates; n=6 for neonates injected with DdCBE-Nd3-9577-1 AAV). Source data are provided as a Source Data file. **b.** Correlation between on-target activity and average mtDNA-wide C·G to T·A off-target editing frequency by the catalytically active DdCBE-Nd3-9577-1 AAV pair in adult mouse heart at 3 weeks or 24 weeks post-injection and in neonatal mouse hearts at 3 weeks post-injection. Source data are provided as a Source Data file.

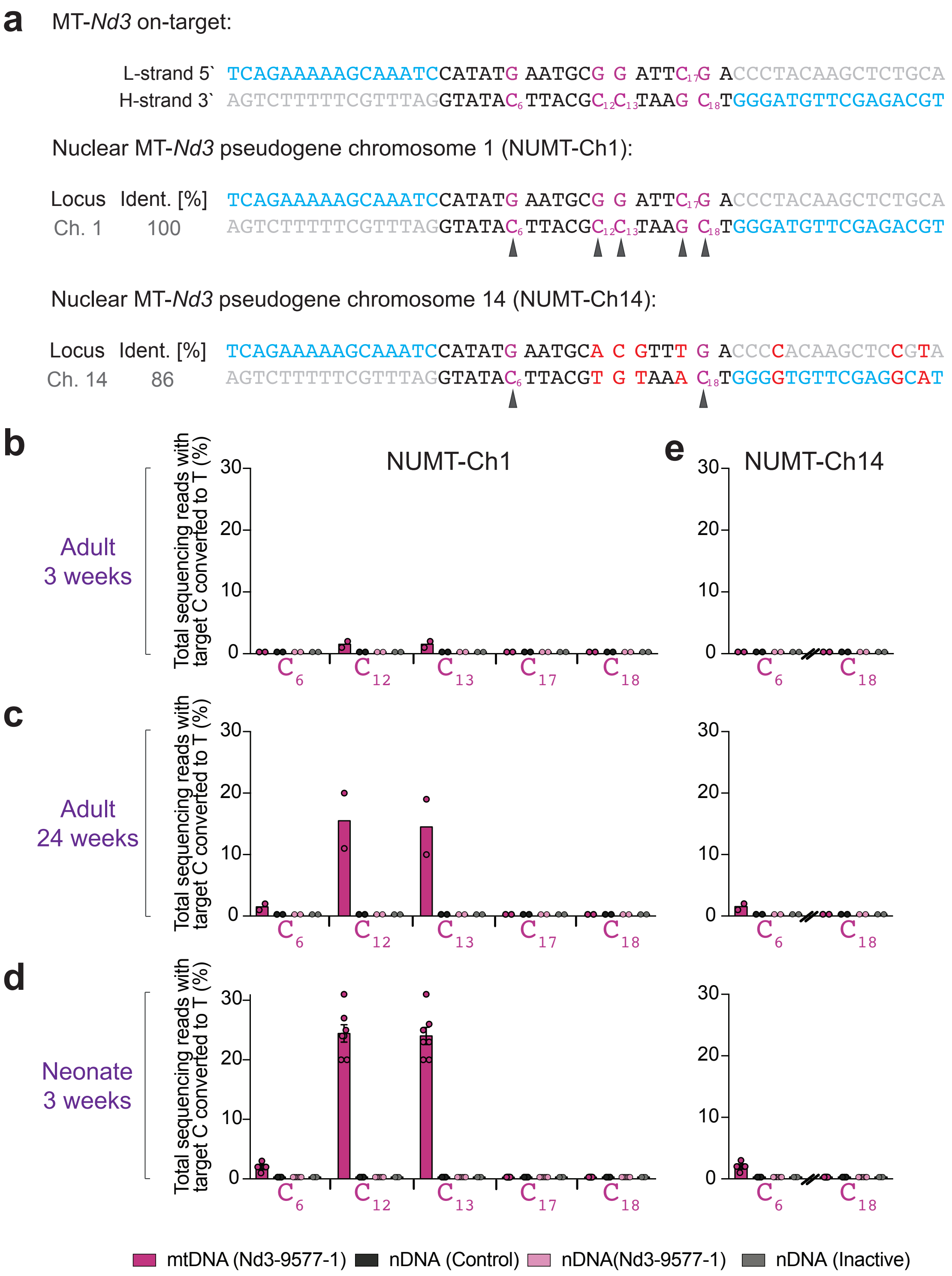

Supplementary Fig. 4 | Assessment of nuclear DNA off-target activity *in vivo* upon delivery of AAV-DdCBEs.

a. Schematic of the DdCBE-Nd3-9577-1 on-target region within the *MT-Nd3* gene and related regions in nuclear DNA bearing greatest homology: nuclear MT-Nd3 pseudogene located on chromosome 1 (NUMT-Ch1) and nuclear MT-Nd3 pseudogene located on chromosome 14 (NUMT-Ch1). Locus and percentage of sequence identity with the canonical site in mtDNA (Ident.) are shown for each nuclear pseudogene. TALE binding sites are presented in blue and targeted Cs are shown in pink. Bases in red indicate divergence from the target sequence. Arrows indicate the mtDNA targeted Cs conserved in the nuclear pseudogenes. **b,c,d.** NGS analysis of nuclear DNA off-target editing frequency of Cs within the nuclear MT-Nd3 pseudogene 1 (NUMT-Ch1) by AAV-DdCBEs in adult mouse heart at 3 weeks (b) or

24 weeks (c) post-injection and in neonatal mouse hearts at 3 weeks (d) post-injection. Each mouse was injected with either the catalytically active or inactive DdCBE-Nd3-9577-1 AAV pair or vehicle (control). The on-target mtDNA editing frequency is shown for each C for comparison. Bars represent the mean and error bars represent ± SEM (n=2 for all experiments in adults; n=3 for vehicle and inactive base editor controls in neonates; n=6 for neonates injected with DdCBE-Nd3-9577-1 AAV). Source data are provided as a Source Data file. **e,f,g.** NGS analysis of nuclear DNA off-target editing frequency of Cs within the nuclear MT-Nd3 pseudogene 2 (NUMT-Ch14) by AAV-DdCBEs in adult mouse heart at 3 weeks (e) or 24 weeks (f) post-injection and in neonatal mouse hearts at 3 weeks (g) post-injection. Each mouse was injected with either the catalytically active or inactive DdCBE-Nd3-9577-1 AAV pair or vehicle (control). The on-target mtDNA editing frequency is shown for each C for comparison. Bars represent the mean and error bars represent ± SEM (n=2 for all experiments in adults; n=3 for vehicle and inactive base editor controls in neonates; n=6 for neonates injected with DdCBE-Nd3-9577-1 AAV). Source data are provided as a Source Data file.

Supplementary Sequences 1 | Amino acid sequences of DdCBEs-Nd3-9577 used in the cell screen.

The halves used to target the L-strand - DdCBE (L) – in the cell screen have the general architecture of (from N- to C-terminus):

SOD2 MTS – 3xHA – TALE – 2aa linker – DddA$_{tox}$ split – 4aa linker – UGI – T2A – GFP*

<u>Targeting: 5´-TCAGAAAAGCAAATC-3´</u>

DdCBE (L) - Nd3-9577 - G1333-N:

MLSRAVCGTSRQLAPVLGYLGSRQKHSLPAGYPYDVPDYAGYPYDVPDYAGYPYDVPDY
AIHGVPMVDLRTLGYSQQQQEKIKPKVRSTVAQHHEALVGHGFTHAHIVALSQHPAALGTV
AVKYQDMIAALPEATHEAIVGVGKQWSGARALEALLTVAGELRGPPGTLDTGQLLKIAKRG
GVTAVEAVHAWRNALTGAPLNLTPEQVVAIASHDGGKQALETVQRLLPVLCQAHGLTPAQ
VVAIASNIGGKQALETVQRLLPVLCQAHGLTPDQVVAIASNNGGKQALETVQRLLPVLCQAH
GLTPEQVVAIASNIGGKQALETVQRLLPVLCQAHGLTPDQVVAIASNIGGKQALETVQRLLP
VLCQAHGLTPDQVVAIASNIGGKQALETVQRLLPVLCQAHGLTPDQVVAIASNIGGKQALET
VQRLLPVLCQAHGLTPDQVVAIASNIGGKQALETVQRLLPVLCQAHGLTPDQVVAIASNNG
GKQALETVQRLLPVLCQAHGLTPEQVVAIASHDGGKQALETVQRLLPVLCQAHGLTPAQVV
AIASNIGGKQALETVQRLLPVLCQAHGLTPDQVVAIASNIGGKQALETVQRLLPVLCQAHGL
TPDQVVAIASNIGGKQALETVQRLLPVLCQAHGLTPDQVVAIASNGGGKQALETVQRLLPVL
CQAHGLTPEQVVAIASHDGGRPALESIVAQLSRPDPALAALTNDHLVALACLGGRPALDAV
KKGLPHAPALIKRTNRRIPERTSHRVAGSGSYALGPYQISAPQLPAYNGQTVGTFYYVNDA
GGLESKVFSSGGSGGSTNLSDIIEKETGKQLVIQESILMLPEEVEEVIGNKPESDILVHTAYD
ESTDENVMLLTSDAPEYKPWALVIQDSNGENKIKMLRSGSGEGRGSLLTCGDVEEN/T2A_c
leavage_site/PGPRLEMVSKGEELFTGVVPILVELDGDVNGHKFSVSGEGEGDATYGKLTLK
FICTTGKLPVPWPTLVTTLTYGVQCFSRYPDHMKQHDFFKSAMPEGYVQERTIFFKDDGNY
KTRAEVKFEGDTLVNRIELKGIDFKEDGNILGHKLEYNYNSHNVYIMADKQKNGIKVNFKIRH
NIEDGSVQLADHYQQNTPIGDGPVLLPDNHYLSTQSALSKDPNEKRDHMVLLEFVTAAGITL
GMDELYK*

DdCBE (L) - Nd3-9577 - G1333-C:

MLSRAVCGTSRQLAPVLGYLGSRQKHSLPAGYPYDVPDYAGYPYDVPDYAGYPYDVPDY
AIHGVPMVDLRTLGYSQQQQEKIKPKVRSTVAQHHEALVGHGFTHAHIVALSQHPAALGTV
AVKYQDMIAALPEATHEAIVGVGKQWSGARALEALLTVAGELRGPPGTLDTGQLLKIAKRG
GVTAVEAVHAWRNALTGAPLNLTPEQVVAIASHDGGKQALETVQRLLPVLCQAHGLTPAQ
VVAIASNIGGKQALETVQRLLPVLCQAHGLTPDQVVAIASNNGGKQALETVQRLLPVLCQAH
GLTPEQVVAIASNIGGKQALETVQRLLPVLCQAHGLTPDQVVAIASNIGGKQALETVQRLLP
VLCQAHGLTPDQVVAIASNIGGKQALETVQRLLPVLCQAHGLTPDQVVAIASNIGGKQALET
VQRLLPVLCQAHGLTPDQVVAIASNIGGKQALETVQRLLPVLCQAHGLTPDQVVAIASNNG
GKQALETVQRLLPVLCQAHGLTPEQVVAIASHDGGKQALETVQRLLPVLCQAHGLTPAQVV
AIASNIGGKQALETVQRLLPVLCQAHGLTPDQVVAIASNIGGKQALETVQRLLPVLCQAHGL
TPDQVVAIASNIGGKQALETVQRLLPVLCQAHGLTPDQVVAIASNGGGKQALETVQRLLPVL
CQAHGLTPEQVVAIASHDGGRPALESIVAQLSRPDPALAALTNDHLVALACLGGRPALDAV
KKGLPHAPALIKRTNRRIPERTSHRVAGSPTPYPNYANAGHVEGQSALFMRDNGISEGLVF
HNNPEGTCGFCVNMTETLLPENAKMTVVPPEGAIPVKRGATGETKVFTGNSNSPKSPTKG
GCSGGSTNLSDIIEKETGKQLVIQESILMLPEEVEEVIGNKPESDILVHTAYDESTDENVMLLT
SDAPEYKPWALVIQDSNGENKIKMLRSGSGEGRGSLLTCGDVEEN/T2A_cleavage_site/PG

PRLEMVSKGEELFTGVVPILVELDGDVNGHKFSVSGEGEGDATYGKLTLKFICTTGKLPVP
WPTLVTTLTYGVQCFSRYPDHMKQHDFFKSAMPEGYVQERTIFFKDDGNYKTRAEVKFEG
DTLVNRIELKGIDFKEDGNILGHKLEYNYNSHNVYIMADKQKNGIKVNFKIRHNIEDGSVQLA
DHYQQNTPIGDGPVLLPDNHYLSTQSALSKDPNEKRDHMVLLEFVTAAGITLGMDELYK*

DdCBE (L) - Nd3-9577 - G1337-N:

MLSRAVCGTSRQLAPVLGYLGSRQKHSLPAGYPYDVPDYAGYPYDVPDYAGYPYDVPDY
AIHGVPMVDLRTLGYSQQQQEKIKPKVRSTVAQHHEALVGHGFTHAHIVALSQHPAALGTV
AVKYQDMIAALPEATHEAIVGVGKQWSGARALEALLTVAGELRGPPGTLDTGQLLKIAKRG
GVTAVEAVHAWRNALTGAPLNLTPEQVVAIASHDGGKQALETVQRLLPVLCQAHGLTPAQ
VVAIASNIGGKQALETVQRLLPVLCQAHGLTPDQVVAIASNNGGKQALETVQRLLPVLCQAH
GLTPEQVVAIASNIGGKQALETVQRLLPVLCQAHGLTPDQVVAIASNIGGKQALETVQRLLP
VLCQAHGLTPDQVVAIASNIGGKQALETVQRLLPVLCQAHGLTPDQVVAIASNIGGKQALET
VQRLLPVLCQAHGLTPDQVVAIASNIGGKQALETVQRLLPVLCQAHGLTPDQVVAIASNNG
GKQALETVQRLLPVLCQAHGLTPEQVVAIASHDGGKQALETVQRLLPVLCQAHGLTPAQVV
AIASNIGGKQALETVQRLLPVLCQAHGLTPDQVVAIASNIGGKQALETVQRLLPVLCQAHGL
TPDQVVAIASNIGGKQALETVQRLLPVLCQAHGLTPDQVVAIASNGGGKQALETVQRLLPVL
CQAHGLTPEQVVAIASHDGGRPALESIVAQLSRPDPALAALTNDHLVALACLGGRPALDAV
KKGLPHAPALIKRTNRRIPERTSHRVAGSGSYALGPYQISAPQLPAYNGQTVGTFYYVNDA
GGLESKVFSSGGPTPYPNYANAGHVEGQSALFMRDNGISEGLVFHNNPEGTCGFCVNMT
ETLLPENAKMTVVPPEGSGGSTNLSDIIEKETGKQLVIQESILMLPEEVEEVIGNKPESDILVH
TAYDESTDENVMLLTSDAPEYKPWALVIQDSNGENKIKMLRSGSGEGRGSLLTCGDVEEN/
T2A_cleavage_site/PGPRLEMVSKGEELFTGVVPILVELDGDVNGHKFSVSGEGEGDATYG
KLTLKFICTTGKLPVPWPTLVTTLTYGVQCFSRYPDHMKQHDFFKSAMPEGYVQERTIFFK
DDGNYKTRAEVKFEGDTLVNRIELKGIDFKEDGNILGHKLEYNYNSHNVYIMADKQKNGIKV
NFKIRHNIEDGSVQLADHYQQNTPIGDGPVLLPDNHYLSTQSALSKDPNEKRDHMVLLEFV
TAAGITLGMDELYK*

DdCBE (L) - Nd3-9577 - G1337-C:

MLSRAVCGTSRQLAPVLGYLGSRQKHSLPAGYPYDVPDYAGYPYDVPDYAGYPYDVPDY
AIHGVPMVDLRTLGYSQQQQEKIKPKVRSTVAQHHEALVGHGFTHAHIVALSQHPAALGTV
AVKYQDMIAALPEATHEAIVGVGKQWSGARALEALLTVAGELRGPPGTLDTGQLLKIAKRG
GVTAVEAVHAWRNALTGAPLNLTPEQVVAIASHDGGKQALETVQRLLPVLCQAHGLTPAQ
VVAIASNIGGKQALETVQRLLPVLCQAHGLTPDQVVAIASNNGGKQALETVQRLLPVLCQAH
GLTPEQVVAIASNIGGKQALETVQRLLPVLCQAHGLTPDQVVAIASNIGGKQALETVQRLLP
VLCQAHGLTPDQVVAIASNIGGKQALETVQRLLPVLCQAHGLTPDQVVAIASNIGGKQALET
VQRLLPVLCQAHGLTPDQVVAIASNIGGKQALETVQRLLPVLCQAHGLTPDQVVAIASNNG
GKQALETVQRLLPVLCQAHGLTPEQVVAIASHDGGKQALETVQRLLPVLCQAHGLTPAQVV
AIASNIGGKQALETVQRLLPVLCQAHGLTPDQVVAIASNIGGKQALETVQRLLPVLCQAHGL
TPDQVVAIASNIGGKQALETVQRLLPVLCQAHGLTPDQVVAIASNGGGKQALETVQRLLPVL
CQAHGLTPEQVVAIASHDGGRPALESIVAQLSRPDPALAALTNDHLVALACLGGRPALDAV
KKGLPHAPALIKRTNRRIPERTSHRVAGSAIPVKRGATGETKVFTGNSNSPKSPTKGGCSG
GSTNLSDIIEKETGKQLVIQESILMLPEEVEEVIGNKPESDILVHTAYDESTDENVMLLTSDAP
EYKPWALVIQDSNGENKIKMLRSGSGEGRGSLLTCGDVEEN/T2A_cleavage_site/PGPRLE
MVSKGEELFTGVVPILVELDGDVNGHKFSVSGEGEGDATYGKLTLKFICTTGKLPVPWPTL
VTTLTYGVQCFSRYPDHMKQHDFFKSAMPEGYVQERTIFFKDDGNYKTRAEVKFEGDTLV
NRIELKGIDFKEDGNILGHKLEYNYNSHNVYIMADKQKNGIKVNFKIRHNIEDGSVQLADHYQ
QNTPIGDGPVLLPDNHYLSTQSALSKDPNEKRDHMVLLEFVTAAGITLGMDELYK*

The halves used to target the H-strand - DdCBE (H) – in the cell screen have the general architecture of (from N- to C-terminus):

SOD2 MTS – 3xHA – TALE – 2aa linker – DddA$_{tox}$ split – 4aa linker – UGI – E2A – RFP*

Targeting: 5´-TGCAGAGCTTGTAGG-3´

DdCBE (H) - Nd3-9577 - G1333-N:

MLSRAVCGTSRQLAPVLGYLGSRQKHSLPAGYPYDVPDYAGYPYDVPDYAGYPYDVPDY
AIHGVPMVDLRTLGYSQQQQEKIKPKVRSTVAQHHEALVGHGFTHAHIVALSQHPAALGTV
AVKYQDMIAALPEATHEAIVGVGKQWSGARALEALLTVAGELRGPPGTLDTGQLLKIAKRG
GVTAVEAVHAWRNALTGAPLNLTPEQVVAIASNNGGKQALETVQRLLPVLCQAHGLTPEQ
VVAIASHDGGKQALETVQRLLPVLCQAHGLTPAQVVAIASNIGGKQALETVQRLLPVLCQAH
GLTPDQVVAIASNNGGKQALETVQRLLPVLCQAHGLTPEQVVAIASNIGGKQALETVQRLLP
VLCQAHGLTPDQVVAIASNNGGKQALETVQRLLPVLCQAHGLTPEQVVAIASHDGGKQALE
TVQRLLPVLCQAHGLTPAQVVAIASNGGGKQALETVQRLLPVLCQAHGLTPEQVVAIASNG
GGKQALETVQRLLPVLCQAHGLTPEQVVAIASNNGGKQALETVQRLLPVLCQAHGLTPEQV
VAIASNGGGKQALETVQRLLPVLCQAHGLTPEQVVAIASNIGGKQALETVQRLLPVLCQAH
GLTPDQVVAIASNNGGKQALETVQRLLPVLCQAHGLTPEQVVAIASNNGGKQALETVQRLL
PVLCQAHGLTPEQVVAIASNNGGRPALESIVAQLSRPDPALAALTNDHLVALACLGGRPALD
AVKKGLPHAPALIKRTNRRIPERTSHRVAGSGSYALGPYQISAPQLPAYNGQTVGTFYYVN
DAGGLESKVFSSGGSGGSTNLSDIIEKETGKQLVIQESILMLPEEVEEVIGNKPESDILVHTA
YDESTDENVMLLTSDAPEYKPWALVIQDSNGENKIKMLRSGQCTNYALLKLAGDVESNPG/
E2A_cleavage_site/PGGGVSKGEELIKENMHMKLYMEGTVNNHHFKCTSEGEGKPYEGTQ
TMRIKVVEGGPLPFAFDILATSFMYGSRTFINHTQGIPDFFKQSFPEGFTWERVTTYEDGGV
LTATQDTSLQDGCLIYNVKIRGVNFPSNGPVMQKKTLGWEANTEMLYPADGGLEGRSDMA
LKLVGGGHLICNFKTTYRSKKPAKNLKMPGVYYVDHRLERIKEADKETYVEQHEVAVARYC
DLPSKLGHKLNKLNPPDESGPGCMSCKCVLS*

DdCBE (H) - Nd3-9577 - G1333-C:

MLSRAVCGTSRQLAPVLGYLGSRQKHSLPAGYPYDVPDYAGYPYDVPDYAGYPYDVPDY
AIHGVPMVDLRTLGYSQQQQEKIKPKVRSTVAQHHEALVGHGFTHAHIVALSQHPAALGTV
AVKYQDMIAALPEATHEAIVGVGKQWSGARALEALLTVAGELRGPPGTLDTGQLLKIAKRG
GVTAVEAVHAWRNALTGAPLNLTPEQVVAIASNNGGKQALETVQRLLPVLCQAHGLTPEQ
VVAIASHDGGKQALETVQRLLPVLCQAHGLTPAQVVAIASNIGGKQALETVQRLLPVLCQAH
GLTPDQVVAIASNNGGKQALETVQRLLPVLCQAHGLTPEQVVAIASNIGGKQALETVQRLLP
VLCQAHGLTPDQVVAIASNNGGKQALETVQRLLPVLCQAHGLTPEQVVAIASHDGGKQALE
TVQRLLPVLCQAHGLTPAQVVAIASNGGGKQALETVQRLLPVLCQAHGLTPEQVVAIASNG
GGKQALETVQRLLPVLCQAHGLTPEQVVAIASNNGGKQALETVQRLLPVLCQAHGLTPEQV
VAIASNGGGKQALETVQRLLPVLCQAHGLTPEQVVAIASNIGGKQALETVQRLLPVLCQAH
GLTPDQVVAIASNNGGKQALETVQRLLPVLCQAHGLTPEQVVAIASNNGGKQALETVQRLL
PVLCQAHGLTPEQVVAIASNNGGRPALESIVAQLSRPDPALAALTNDHLVALACLGGRPALD
AVKKGLPHAPALIKRTNRRIPERTSHRVAGSPTPYPNYANAGHVEGQSALFMRDNGISEGL
VFHNNPEGTCGFCVNMTETLLPENAKMTVVPPEGAIPVKRGATGETKVFTGNSNSPKSPT
KGGCSGGSTNLSDIIEKETGKQLVIQESILMLPEEVEEVIGNKPESDILVHTAYDESTDENVM
LLTSDAPEYKPWALVIQDSNGENKIKMLRSGQCTNYALLKLAGDVESNPG/E2A_cleavage_
site/PGGGVSKGEELIKENMHMKLYMEGTVNNHHFKCTSEGEGKPYEGTQTMRIKVVEGGP
LPFAFDILATSFMYGSRTFINHTQGIPDFFKQSFPEGFTWERVTTYEDGGVLTATQDTSLQD
GCLIYNVKIRGVNFPSNGPVMQKKTLGWEANTEMLYPADGGLEGRSDMALKLVGGGHLIC
NFKTTYRSKKPAKNLKMPGVYYVDHRLERIKEADKETYVEQHEVAVARYCDLPSKLGHKLN
KLNPPDESGPGCMSCKCVLS*

DdCBE (H) - Nd3-9577 - G1337-N:

MLSRAVCGTSRQLAPVLGYLGSRQKHSLPAGYPYDVPDYAGYPYDVPDYAGYPYDVPDY
AIHGVPMVDLRTLGYSQQQQEKIKPKVRSTVAQHHEALVGHGFTHAHIVALSQHPAALGTV
AVKYQDMIAALPEATHEAIVGVGKQWSGARALEALLTVAGELRGPPGTLDTGQLLKIAKRG
GVTAVEAVHAWRNALTGAPLNLTPEQVVAIASNNGGKQALETVQRLLPVLCQAHGLTPEQ
VVAIASHDGGKQALETVQRLLPVLCQAHGLTPAQVVAIASNIGGKQALETVQRLLPVLCQAH
GLTPDQVVAIASNNGGKQALETVQRLLPVLCQAHGLTPEQVVAIASNIGGKQALETVQRLLP
VLCQAHGLTPDQVVAIASNNGGKQALETVQRLLPVLCQAHGLTPEQVVAIASHDGGKQALE
TVQRLLPVLCQAHGLTPAQVVAIASNGGGKQALETVQRLLPVLCQAHGLTPEQVVAIASNG
GGKQALETVQRLLPVLCQAHGLTPEQVVAIASNNGGKQALETVQRLLPVLCQAHGLTPEQV
VAIASNGGGKQALETVQRLLPVLCQAHGLTPEQVVAIASNIGGKQALETVQRLLPVLCQAH
GLTPDQVVAIASNNGGKQALETVQRLLPVLCQAHGLTPEQVVAIASNNGGKQALETVQRLL
PVLCQAHGLTPEQVVAIASNNGGRPALESIVAQLSRPDPALAALTNDHLVALACLGGRPALD
AVKKGLPHAPALIKRTNRRIPERTSHRVAGSGSYALGPYQISAPQLPAYNGQTVGTFYYVN
DAGGLESKVFSSGGPTPYPNYANAGHVEGQSALFMRDNGISEGLVFHNNPEGTCGFCVN
MTETLLPENAKMTVVPPEGSGGSTNLSDIIEKETGKQLVIQESILMLPEEVEEVIGNKPESDIL
VHTAYDESTDENVMLLTSDAPEYKPWALVIQDSNGENKIKMLRSGQCTNYALLKLAGDVES
NPG/E2A_cleavage_site/PGGGVSKGEELIKENMHMKLYMEGTVNNHHFKCTSEGEGKPYE
GTQTMRIKVVEGGPLPFAFDILATSFMYGSRTFINHTQGIPDFFKQSFPEGFTWERVTTYED
GGVLTATQDTSLQDGCLIYNVKIRGVNFPSNGPVMQKKTLGWEANTEMLYPADGGLEGRS
DMALKLVGGGHLICNFKTTYRSKKPAKNLKMPGVYYVDHRLERIKEADKETYVEQHEVAVA
RYCDLPSKLGHKLNKLNPPDESGPGCMSCKCVLS*

DdCBE (H) - Nd3-9577 - G1337-C:

MLSRAVCGTSRQLAPVLGYLGSRQKHSLPAGYPYDVPDYAGYPYDVPDYAGYPYDVPDY
AIHGVPMVDLRTLGYSQQQQEKIKPKVRSTVAQHHEALVGHGFTHAHIVALSQHPAALGTV
AVKYQDMIAALPEATHEAIVGVGKQWSGARALEALLTVAGELRGPPGTLDTGQLLKIAKRG
GVTAVEAVHAWRNALTGAPLNLTPEQVVAIASNNGGKQALETVQRLLPVLCQAHGLTPEQ
VVAIASHDGGKQALETVQRLLPVLCQAHGLTPAQVVAIASNIGGKQALETVQRLLPVLCQAH
GLTPDQVVAIASNNGGKQALETVQRLLPVLCQAHGLTPEQVVAIASNIGGKQALETVQRLLP
VLCQAHGLTPDQVVAIASNNGGKQALETVQRLLPVLCQAHGLTPEQVVAIASHDGGKQALE
TVQRLLPVLCQAHGLTPAQVVAIASNGGGKQALETVQRLLPVLCQAHGLTPEQVVAIASNG
GGKQALETVQRLLPVLCQAHGLTPEQVVAIASNNGGKQALETVQRLLPVLCQAHGLTPEQV
VAIASNGGGKQALETVQRLLPVLCQAHGLTPEQVVAIASNIGGKQALETVQRLLPVLCQAH
GLTPDQVVAIASNNGGKQALETVQRLLPVLCQAHGLTPEQVVAIASNNGGKQALETVQRLL
PVLCQAHGLTPEQVVAIASNNGGRPALESIVAQLSRPDPALAALTNDHLVALACLGGRPALD
AVKKGLPHAPALIKRTNRRIPERTSHRVAGSAIPVKRGATGETKVFTGNSNSPKSPTKGGC
SGGSTNLSDIIEKETGKQLVIQESILMLPEEVEEVIGNKPESDILVHTAYDESTDENVMLLTSD
APEYKPWALVIQDSNGENKIKMLRSGQCTNYALLKLAGDVESNPG/E2A_cleavage_site/PG
GGVSKGEELIKENMHMKLYMEGTVNNHHFKCTSEGEGKPYEGTQTMRIKVVEGGPLPFAF
DILATSFMYGSRTFINHTQGIPDFFKQSFPEGFTWERVTTYEDGGVLTATQDTSLQDGCLIY
NVKIRGVNFPSNGPVMQKKTLGWEANTEMLYPADGGLEGRSDMALKLVGGGHLICNFKTT
YRSKKPAKNLKMPGVYYVDHRLERIKEADKETYVEQHEVAVARYCDLPSKLGHKLNKLNPP
DESGPGCMSCKCVLS*

Supplementary Sequences 2 | Amino acid sequences of DdCBEs-Nd3-9577 used in the AAV experiments.

The halves used to target the L-strand - DdCBE (L) – in the AAV experiments are similar to the cell screen, except that the T2A self-cleavage peptide and the fluorescent marker GFP were substituted by a stop codon at the end of UGI, resulting in the architecture of (from N- to C-terminus):

SOD2 MTS – 3xHA – TALE – 2aa linker – $DddA_{tox}$ G1333-C – 4aa linker – UGI*

Targeting: 5´-TCAGAAAAGCAAATC-3´

AAV- DdCBE (L) - Nd3-9577 - G1333-C:

MLSRAVCGTSRQLAPVLGYLGSRQKHSLPAGYPYDVPDYAGYPYDVPDYAGYPYDVPDY
AIHGVPMVDLRTLGYSQQQQEKIKPKVRSTVAQHHEALVGHGFTHAHIVALSQHPAALGTV
AVKYQDMIAALPEATHEAIVGVGKQWSGARALEALLTVAGELRGPPGTLDTGQLLKIAKRG
GVTAVEAVHAWRNALTGAPLNLTPEQVVAIASHDGGKQALETVQRLLPVLCQAHGLTPAQ
VVAIASNIGGKQALETVQRLLPVLCQAHGLTPDQVVAIASNNGGKQALETVQRLLPVLCQAH
GLTPEQVVAIASNIGGKQALETVQRLLPVLCQAHGLTPDQVVAIASNIGGKQALETVQRLLP
VLCQAHGLTPDQVVAIASNIGGKQALETVQRLLPVLCQAHGLTPDQVVAIASNIGGKQALET
VQRLLPVLCQAHGLTPDQVVAIASNIGGKQALETVQRLLPVLCQAHGLTPDQVVAIASNNG
GKQALETVQRLLPVLCQAHGLTPEQVVAIASHDGGKQALETVQRLLPVLCQAHGLTPAQVV
AIASNIGGKQALETVQRLLPVLCQAHGLTPDQVVAIASNIGGKQALETVQRLLPVLCQAHGL
TPDQVVAIASNIGGKQALETVQRLLPVLCQAHGLTPDQVVAIASNGGGKQALETVQRLLPVL
CQAHGLTPEQVVAIASHDGGRPALESIVAQLSRPDPALAALTNDHLVALACLGGRPALDAV
KKGLPHAPALIKRTNRRIPERTSHRVAGSPTPYPNYANAGHVEGQSALFMRDNGISEGLVF
HNNPEGTCGFCVNMTETLLPENAKMTVVPPEGAIPVKRGATGETKVFTGNSNSPKSPTKG
GCSGGSTNLSDIIEKETGKQLVIQESILMLPEEVEEVIGNKPESDILVHTAYDESTDENVMLLT
SDAPEYKPWALVIQDSNGENKIKML*

The halves used to target the H-strand - DdCBE (H) – in the AAV experiments are similar to the cell screen, except that the E2A self-cleavage peptide and the fluorescent marker RFP were substituted by a stop codon at the end of UGI and the 3xHA tag substituted by a 3xFLAG tag, resulting in the architecture of (from N- to C-terminus):

SOD2 MTS – 3xFLAG – TALE – 2aa linker – $DddA_{tox}$ G1333-N – 4aa linker – UGI*

Targeting: 5´-TGCAGAGCTTGTAGG-3´

AAV- DdCBE (H) - Nd3-9577 - G1333-N:

MLSRAVCGTSRQLAPVLGYLGSRQKHSLPAGDYKDHDGDYKDHDIDYKDDDDKIHGVPMV
DLRTLGYSQQQQEKIKPKVRSTVAQHHEALVGHGFTHAHIVALSQHPAALGTVAVKYQDMI
AALPEATHEAIVGVGKQWSGARALEALLTVAGELRGPPGTLDTGQLLKIAKRGGVTAVEAV
HAWRNALTGAPLNLTPEQVVAIASNNGGKQALETVQRLLPVLCQAHGLTPEQVVAIASHDG
GKQALETVQRLLPVLCQAHGLTPAQVVAIASNIGGKQALETVQRLLPVLCQAHGLTPDQVV

AIASNNGGKQALETVQRLLPVLCQAHGLTPEQVVAIASNIGGKQALETVQRLLPVLCQAHGL
TPDQVVAIASNNGGKQALETVQRLLPVLCQAHGLTPEQVVAIASHDGGKQALETVQRLLPV
LCQAHGLTPAQVVAIASNGGGKQALETVQRLLPVLCQAHGLTPEQVVAIASNGGGKQALET
VQRLLPVLCQAHGLTPEQVVAIASNNGGKQALETVQRLLPVLCQAHGLTPEQVVAIASNGG
GKQALETVQRLLPVLCQAHGLTPEQVVAIASNIGGKQALETVQRLLPVLCQAHGLTPDQVV
AIASNNGGKQALETVQRLLPVLCQAHGLTPEQVVAIASNNGGKQALETVQRLLPVLCQAHG
LTPEQVVAIASNNGGRPALESIVAQLSRPDPALAALTNDHLVALACLGGRPALDAVKKGLPH
APALIKRTNRRIPERTSHRVAGSGSYALGPYQISAPQLPAYNGQTVGTFYYVNDAGGLESK
VFSSGGSGGSTNLSDIIEKETGKQLVIQESILMLPEEVEEVIGNKPESDILVHTAYDESTDEN
VMLLTSDAPEYKPWALVIQDSNGENKIKML*
