# Supplementary material for: In vivo mitochondrial base editing via adeno-associated viral delivery to mouse post-mitotic tissue
Source: Nat Commun. 2022 Feb 8;13:750. doi: 10.1038/s41467-022-28358-w (PMC8825850; doi:10.1038/s41467-022-28358-w)
Supplement: Supplementary file 2 — Reporting Summary [file 41467_2022_28358_MOESM2_ESM.pdf]

nature portfolio

Corresponding author(s): Michal Minczuk

Last updated by author(s): Jan 12, 2022

Reporting Summary

Nature Portfolio wishes to improve the reproducibility of the work that we publish. This form provides structure for consistency and transparency in reporting. For further information on Nature Portfolio policies, see our Editorial Policies and the Editorial Policy Checklist.

Statistics

For all statistical analyses, confirm that the following items are present in the figure legend, table legend, main text, or Methods section.

n/a	Confirmed	
☐	☒	The exact sample size (*n*) for each experimental group/condition, given as a discrete number and unit of measurement
☐	☒	A statement on whether measurements were taken from distinct samples or whether the same sample was measured repeatedly
☐	☒	The statistical test(s) used AND whether they are one- or two-sided<br>*Only common tests should be described solely by name; describe more complex techniques in the Methods section.*
☒	☐	A description of all covariates tested
☒	☐	A description of any assumptions or corrections, such as tests of normality and adjustment for multiple comparisons
☐	☒	A full description of the statistical parameters including central tendency (e.g. means) or other basic estimates (e.g. regression coefficient) AND variation (e.g. standard deviation) or associated estimates of uncertainty (e.g. confidence intervals)
☐	☒	For null hypothesis testing, the test statistic (e.g. *F*, *t*, *r*) with confidence intervals, effect sizes, degrees of freedom and *P* value noted<br>*Give P values as exact values whenever suitable.*
☒	☐	For Bayesian analysis, information on the choice of priors and Markov chain Monte Carlo settings
☒	☐	For hierarchical and complex designs, identification of the appropriate level for tests and full reporting of outcomes
☒	☐	Estimates of effect sizes (e.g. Cohen's *d*, Pearson's *r*), indicating how they were calculated

Our web collection on statistics for biologists contains articles on many of the points above.

Software and code

Policy information about availability of computer code

Data collection: Illumina MiSeq manufacturer's software (version 4.0)

Data analysis:
TrimGalore! version 0.6.6 (doi: 10.5281/zenodo.5127899)
https://github.com/FelixKrueger/TrimGalore/releases
Bowtie version 2 (doi: 10.1038%2Fnmeth.1923)
https://github.com/BenLangmead/bowtie2
Samtools version 1.12 (doi: 10.1093/gigascience/giab008)
https://github.com/samtools/samtools
VarScan version 2 (doi: 10.1101/gr.129684.111)
https://github.com/Jeltje/varscan2
Cutadapt version 3.5 (doi: 10.14806/ej)
https://github.com/marcelm/cutadapt
REDItools 2.0 (doi.org/10.1186/s12859-020-03562-x)
https://github.com/tizianoflati/reditools2.0
ImageJ version 2.1.o/1.53c
Graphpad Prism 9 for macOS version 9.1.0
Microsoft Excel version 15.32

For manuscripts utilizing custom algorithms or software that are central to the research but not yet described in published literature, software must be made available to editors and reviewers. We strongly encourage code deposition in a community repository (e.g. GitHub). See the Nature Portfolio guidelines for submitting code & software for further information.

Data

Policy information about availability of data

All manuscripts must include a data availability statement. This statement should provide the following information, where applicable:

- Accession codes, unique identifiers, or web links for publicly available datasets
- A description of any restrictions on data availability
- For clinical datasets or third party data, please ensure that the statement adheres to our policy

The authors declare that the data supporting the findings of this study are available within the paper and its supplementary information files, apart from proprietary scripts which are available upon request. Source data are provided with this paper. The NGS files have been deposited in GEO: GSE184064.

Field-specific reporting

Please select the one below that is the best fit for your research. If you are not sure, read the appropriate sections before making your selection.

☒ Life sciences ☐ Behavioural & social sciences ☐ Ecological, evolutionary & environmental sciences

For a reference copy of the document with all sections, see nature.com/documents/nr-reporting-summary-flat.pdf

Life sciences study design

All studies must disclose on these points even when the disclosure is negative.

Sample size	No statistical methods were used to predetermine sample size for in vitro and in vivo experiment. Sample sizes were chosen based on existing procedures and standards in the field. Sample size was chosen based on biological replicates, in order to achieve a n at least of 2. Whenever experimentally possible and reasonable, more replicates were performed. In adult-injected mice, n =2 was used as the readout was presence or absence of DNA editing and to comply with the "reduction" stated on the 3Rs principle of animal research. In newborn-injected mice, we injected more animals (>5) to account for possible rejection of the pups by the mother after injection.
Data exclusions	No data was excluded from analysis.
Replication	Biological duplicate or triplicate of cells experiments were done with distinct aliquots of cells at intervals of at least one week. A biological replicate in in vivo experiments corresponds to an individual mouse. All experiments were repeated at least once. All attempts were successful.
Randomization	Adult mice were injected in their numerical order based on their "mouse ID" which is randomly attributted by animal technicians upon arrival in the animal facility. Newborn animals were also injected randomly on a "first come, first served" basis when collecting them from the cage.
Blinding	Tissue samples were collected and analysed by independent researchers, without any description.

Reporting for specific materials, systems and methods

We require information from authors about some types of materials, experimental systems and methods used in many studies. Here, indicate whether each material, system or method listed is relevant to your study. If you are not sure if a list item applies to your research, read the appropriate section before selecting a response.

Materials & experimental systems

n/a	Involved in the study	
☐	☒	Antibodies
☐	☒	Eukaryotic cell lines
☒	☐	Palaeontology and archaeology
☐	☒	Animals and other organisms
☒	☐	Human research participants
☒	☐	Clinical data
☒	☐	Dual use research of concern

Methods

n/a	Involved in the study	
☒	☐	ChIP-seq
☒	☐	Flow cytometry
☒	☐	MRI-based neuroimaging

Antibodies

Antibodies used	Primary antibodies used:<br>1. Rat Anti-HA-tag (clone 3F10) (Roche, 11867423001) - Western Blot<br>2. Mouse Anti-FLAG-M2-tag (clone M2) (Sigma-Aldrich, F3165) - Western Blot<br>3. Rabbit Anti-HA-Tag (Cell Signaling, 3724) - Immunohistochemistry

	Secondary antibodies used:<br>4. HRP-linked Goat Anti-Rat IgG (Cell Signaling, 7077S) - Western blot<br>5. HRP-linked Goat Anti-Mouse IgG (Promega, W4021) - Western blot<br>6. Goat Anti-rabbit IgG Alexa Fluor 594 (Thermofisher, A-11012) - Immunohistochemistry
Validation	Primary antibodies used:<br>1. Rat Anti-HA-tag (clone 3F10) (Roche, 11867423001) - Function tested in western blot by the supplier.<br>2. Mouse Anti-FLAG-M2-tag (Sigma-Aldrich, F3165) - Validated by supplier: "Detects a single band of protein on a western blot from an E. coli crude cell lysate".<br>3. Rabbit Anti-HA-Tag (Cell Signaling, 3724) - Approved for immunohistochemistry by the supplier. No reactivity was detected by us in mouse heart samples of WT animals.

Eukaryotic cell lines

Policy information about cell lines

Cell line source(s)	NIH/3T3 (ATCC; CRL-1658)
Authentication	Cell line was not authenticated.
Mycoplasma contamination	Cell medium was tested for mycoplasma contamination. All tests were negative.
Commonly misidentified lines<br>(See ICLAC register)	None used.

Animals and other organisms

Policy information about studies involving animals; ARRIVE guidelines recommended for reporting animal research

Laboratory animals	Mice (Mus Musculus) in a C57BL/6J background were obtained from Charles River Laboratories. The animals were maintained in a temperature- and humidity-controlled animal care facility with a 12-h light/12-h dark cycle and free access to water and food, and they were sacrificed by cervical dislocation. In adult experiments, we used male mice with 8 weeks of age at the time of injection. In newborn experiments, pups (males and females) were injected at Postnatal day 1.
Wild animals	The study did not involve wild animals.
Field-collected samples	The study did not involve samples collected from the field.
Ethics oversight	All animal experiments were carried out in accordance with the UK Animals (Scientific Procedures) Act 1986 (PPL: P6C20975A) and EU Directive 2010/63/EU.

Note that full information on the approval of the study protocol must also be provided in the manuscript.
